# Green Finance and Technological Innovation in Heavily Polluting Enterprises: Evidence from China

**DOI:** 10.3390/ijerph20043333

**Published:** 2023-02-14

**Authors:** Bingwen Wang, Chen Wang

**Affiliations:** 1 Graduate School, Anhui University of Finance & Economics, Bengbu 233030, China; 2 School of Economics, Anhui University, Hefei 230601, China

**Keywords:** environmental pollution, green finance, technological innovation, green growth, heavily polluting enterprise, environmental regulation

## Abstract

There is an urgent need for countries worldwide to promote the green transformation of their economies and reduce environmental pollution. Based on China’s Green Credit Guidelines policy in 2012 and the data of Chinese listed companies from 2007 to 2021, we conducted an empirical test using the difference-in-differences method. The results showed that green finance policies inhibit technological innovation in heavily polluting enterprises, and the stronger the enterprise’s operating capacity, the weaker this inhibiting effect. The study also shows that bank loan, loan term, corporate management motivation, and business confidence have intermediation effects. Therefore, countries should improve green financial policies and promote technological innovation in heavily polluting enterprises in order to reduce environmental pollution and promote green growth.

## 1. Introduction

There have been increasing concerns on the issue of environmental pollution [1]. To solve the environmental pollution problem, countries around the world are vigorously promoting a green transformation of their economies [2]. In this context, green growth is gaining attention worldwide. Green growth is defined as resilient, clean, and energy-efficient economic growth and is centred on the reconciliation of economic growth with the ecological environment [3].

Technological innovation plays a key role in solving environmental problems and promoting green growth. The importance of technological innovation in facilitating production transformation and increasing the total factor productivity of firms has been repeatedly highlighted by scholars [4]. Long-term solutions to environmental problems require technological innovation, especially green innovation [5]. Khan et al. [6] found that green innovation contributed to green growth by reducing CO_2_ emissions and promoting economic and industrial structural transformation using data from G7 countries. Danish and Ulucak [7] found that environmental technology innovation significantly contributed to green growth in Brazil, Russia, India, China, and South Africa, collectively known as BRICS countries.

Green finance contributes to environmental protection and economic growth. On the one hand, financial development can mobilise savings, promote capital accumulation, and improve capital allocation, and thus contribute to economic growth [8]. On the other hand, financial development can reduce greenhouse gas emissions and contribute to environmental protection [9]. As a financial innovation, green finance is essentially the same as traditional finance, with the main difference being that green finance has an environmental purpose [10]. Green finance provides support for environmental protection and pollution reduction, through financial instruments and products, such as credit, insurance, and securities [11].

The relationship between green finance and technological innovation has an impact on whether green finance policies and technological innovation can be fully effective and is important for solving the environmental pollution problem and promoting green growth. China has implemented green finance policies and is committed to promoting green growth [12]. The Chinese government issued the ‘Green Credit Guidelines’ (hereinafter referred to as ‘the Guidelines’) on 29 January 2012, which explicitly require banking financial institutions to adjust their credit to address environmental risks and support green growth. As banks dominate the Chinese financial system, green credit plays an important part in China’s green finance [13].

Therefore, we regarded the release of the Guidelines as the implementation of green financial policy and, using the data of Chinese listed companies from 2007 to 2021, explored the relationship between green finance and technological innovation. The innovations of this study are as follows: First, this study identified the heterogeneous effects of green finance policies on technological innovation. Second, this study indicated the crucial role of policy signal in the influence of green finance on technological innovation. Third, this study determined the influencing factors of banks and enterprises.

The remainder of the paper is organised as follows: Section 2 and Section 3 review the literature and analyse the interaction between green finance and technological innovation in theoretical terms, respectively. Section 4 and Section 5 introduce the empirical model and report the empirical results, respectively. Section 6 and Section 7 discuss the mediating effects and moderating effects, respectively. Finally, Section 8 presents the conclusions. 

## 2. Research Review

### 2.1. Micro Effects of Green Finance

Relevant literature mainly includes the environmental and technological innovation effects of green finance. Regarding environmental effects, green finance promotes a greener industrial structure and guides enterprises to change their traditional production methods in favour of clean production [14], and thus contributes to environmental protection [15]. In terms of the technological innovation effects of green finance, related studies have come to different conclusions. Yu et al. [16] argued that green finance facilitates technological innovation by providing financing support. However, Andersen [17] argued that green finance can strengthen finance constraints and stimulate companies to invest in tangible assets, and thus reduce research and development (R&D) investment.

Financial markets can promote technological innovation by efficiently allocating financial resources and sharing the risk of R&D [18]. Conversely, the technological innovation of a firm is limited if they lack financial support and face financial constraints [19]. Financial constraints have different effects on companies abandoning innovation projects at different stages of their implementation, and in the project justification stage, financial constraints have the greatest influence on a firm’s abandonment of innovation investments [20]. 

### 2.2. Environmental Regulation and Technological Innovation

Some scholars believe that environmental regulation inevitably increases firms’ running costs, and therefore crowds out their investments in technological innovation and diminishes their advantages [21]. In contrast, Porter and Van [22] showed that while environmental regulations increase firms’ costs, they also provide incentives for firms to innovate, and thus enhances their competitiveness and compensates for the increased production costs. This results in an innovation compensation effect. Therefore, environmental regulation significantly contributed to technological innovation [23].

### 2.3. Research Gaps

From the above literature, it can be seen there is a paucity of literature that directly studies the interactions between green finance and technological innovation. The existing studies on this topic suffer from the following limitations: First, the studies are on green innovation rather than technological innovation. Technological innovation includes not only green innovation but also other technological innovation such as innovation to improve productivity. Secondly, the research findings are controversial. Thirdly, there is a lack of in-depth and comprehensive analysis of the mechanism of action between green finance and technological innovation. Therefore, there is a need for further research on this topic.

## 3. Theoretical Analysis

### 3.1. Technological Innovation and Green Finance

In this study, the compliance cost effects, innovation compensation effects, and policy signalling effects of green financial policies were analysed.

As a market-led environmental regulation policy, the Guidelines require banks to use firms’ environmental performance as an important basis for credit allocation, thus prompting firms to increase their environmental expenditures to obtain loans. Given the limited resources, the increase in environmental protection costs may crowd-out funds for other production activities, including R&D. Therefore, the Guidelines may have a ‘cost compliance effect’, creating a disincentive for technological innovation. In contrast, the Guidelines increase the environmental costs of obtaining credit, thereby increasing cost pressures on firms. Under this pressure, firms may seek technological innovations to improve productivity and thus compensate for the increased environmental costs. Thus, the Guidelines may have an ‘innovation compensation effect’ and promote technological innovation.

As R&D activities are characterised by long investment cycles, high capital requirements, and uncertain outcomes [24], sustained and sufficient R&D investment is required to generate applicable technological innovations. If the market environment deteriorates in the future and a company cannot continue to invest in R&D and does not achieve an applicable technological innovation, then the initial R&D investment will become a sunk cost. In a deteriorating market environment, even if the R&D investment is successful, the products will still face sale problems and it will be difficult to translate into a competitive advantage for the company. Therefore, under the pressure of increasing environmental costs, it is in the interest of companies to invest technological innovation only if the industry has a broad market space in the future. However, the introduction of the Guidelines has signalled that the government will tighten restrictions on heavily polluting industries and enterprises. These industries are pessimistic about whether R&D investment will bring effective ‘innovation compensation’. 

In general, the ‘compliance cost effect’ of green finance policies can inhibit technological innovation, while the ‘innovation compensation effect’ promotes it. The ‘policy signalling effect’ inhibits the ‘innovation compensation effect’. Therefore, green finance policies generally discourage technological innovation.

Accordingly, we proposed Hypothesis 1:

**Hypothesis 1** **(H1).** *Green financial policies in general behave as a disincentive to technological innovation*.

### 3.2. Mechanisms by Which Green Financial Policies Affect Technological Innovation

#### 3.2.1. Bank Loan Intermediation Effects

The Guidelines require banking financial institutions to develop specific credit policies for restricted industries, such as heavily polluting industries, and to deny credit for non-compliance with environmental performance. This reduces the lending banks do to heavily polluting industries. There is a problem of excessive credit in China’s heavily polluting industries [25], and a reduction in bank lending could weaken banks’ restrictions on technological innovation, and thus increase innovation. Banks lend to firms at a fixed interest rate based on the amount of the loan, and even if the firm succeeds in technological innovation and achieves greater economic benefits, the bank does not receive a share of the additional revenue. If a company fails to innovate and has serious financial problems, bank loans are at risk. Therefore, banks tend to restrict firms’ innovative behaviour [26].

Accordingly, we proposed Hypothesis 2:

**Hypothesis 2** **(H2).** *Green financial policies promote technological innovation by reducing bank loans and weakening constraints on technological innovation of companies*.

#### 3.2.2. Loan Term Intermediation Effects

In the case of short loan terms, changes in business conditions and market demand are small and loans can be recovered; therefore, the loan risk is manageable. However, for longer loan terms, the business conditions, market demand, and policy direction may change significantly, and the loan risk is more variable. The policy signals released by the Guidelines have made banks aware of the potential for further tightening of policy restrictions on heavily polluting industries in the future, which could increase the financial risk of enterprises and the associated credit risk for banks. Therefore, under the influence of the Guidelines, banks have tended to shorten the maturity of credit to hedge potential policy risks. As the maturity structure of debt needs to match the asset structure [27] and technological innovation requires long-term funding, shorter credit maturities may discourage technological innovation.

Accordingly, we proposed Hypothesis 3:

**Hypothesis 3** **(H3).** *Green financial policies discourage technological innovation by shortening the term of loans.*

#### 3.2.3. Management Motivation Mediating Effects

The implementation of the Guidelines has prompted banks to strengthen their supervision of companies and reduce the incentive of their management. First, banks have strengthened their oversight of corporate environmental practices. The Guidelines require banking institutions to dynamically assess and classify the environmental risks of their clients, with the relevant results serving as an important basis for their ratings and credit access. Second, banks have strengthened their supervision of business operations. Banks are an important player in corporate governance [28] and can monitor and influence the operations of borrowers. Green finance policies increase the environmental costs and release policy signals that are unfavourable to traditional companies, and thus increase the risk of bank loans. Banks will inevitably strengthen their supervision of the business management process and increase restrictions on corporate behaviour, which will reduce room for autonomous decision-making by corporate management and reduce motivation. As R&D innovation is characterised by long investment cycles, large amounts of money, and high risks, the demonstration and implementation of R&D projects require professional management skills and corresponding decision-making responsibilities. Therefore, if management loses motivation, they will reduce technological innovation activities.

Accordingly, we proposed Hypothesis 4:

**Hypothesis 4** **(H4).** *Green financial policies reduce the scope for corporate autonomy and reduce the incentive of corporate management, and thereby discourage technological innovation*.

#### 3.2.4. Business Confidence Mediating Effects

Business confidence is a composite judgement made by firms based on their understanding of the current situation and economic information [29]. Government support can provide protection for business development and financial support can provide funding for business development, both of which have a significant impact on business confidence. The introduction of the Guidelines indicates the government’s desire to restrict heavily polluting industries and use financial instruments to enhance the financing constraints of enterprises. These changes will inevitably lead to a decline in the confidence of enterprises in heavily polluting industries. When enterprises are pessimistic, they generally have ‘high risk, low return’ expectations of investment projects and tend to reduce their investment [30].

Accordingly, we proposed Hypothesis 5:

**Hypothesis 5** **(H5).** *Green financial policies discourage technological innovation by weakening business confidence.*

### 3.3. Moderating Mechanisms for Green Financial Policies to Influence Technological Innovation

Operational capability indicates the efficiency of an enterprise’s use of resources to achieve its management objectives. First, companies with strong operational capabilities can allocate their assets in a rational manner, and thus can reduce the crowding out of R&D investment by environmental costs and focus more resources on R&D. Second, in the face of a deteriorating market environment, companies with strong operational capabilities are more confident of surviving the strong market competition in the future and therefore have a greater willingness to engage in technological innovation.

Accordingly, we proposed Hypothesis 6:

**Hypothesis 6** **(H6).** *The stronger the operational capacity of the enterprise, the weaker the inhibitory performance of green financial policies on technological innovation*.

## 4. Empirical Design

### 4.1. Data and Sample

In this study, an empirical test was conducted using data from Chinese listed companies for the period 2007–2021. The Wind and CSMAR databases provided data to support this study. The following companies were excluded: financial companies, companies with unusual transactions, insolvent companies, and data deficient companies. The continuous variables were tail shrunk to avoid non-normal data effects. Finally, we obtained 19,469 annual observations.

### 4.2. Model Construction and Variable Definition

The model is as follows:

(1) $$\mathrm{RD}\_{\mathrm{asse}}_{\mathrm{it}}=\alpha_{0}+\alpha_{1}{\mathrm{Pollu}}_{i}\times {\mathrm{Policy}}_{t}+\alpha_{2}X_{\mathrm{it}}+\varphi_{i}+\varphi_{t}+\varepsilon_{\mathrm{it}}$$

where t and i denote the year and company, respectively; $\mathrm{RD}\_{\mathrm{asse}}_{\mathrm{it}}$ denotes enterprise i’s innovation input in year t; ${\mathrm{Pollu}}_{i}$ denotes the dummy variable for companies in heavily polluting industries; ${\mathrm{Policy}}_{t}$ denotes the dummy variable for years in which the Guidelines worked; ${\;X}_{\mathrm{it}}$ denotes control variables; $\varphi_{t}$ and $\varphi_{i}$ denote year and firm fixed effects, respectively; and $\varepsilon_{\mathrm{it}}$ denotes the random error.

#### 4.2.1. Dependent Variables

Commonly used indicators for measuring technological innovation are innovation inputs and outputs, with the former generally using R&D input indicators and the latter generally using patent application indicators. This study focused on whether an enterprise’s innovation activities are more active. Thus, R&D input indicators can indicate a company’s technological innovation accurately, and in a timely manner. Specifically, the dependent variable of the empirical model is R&D expenditure as a percentage of total assets.

#### 4.2.2. Independent Variables

When the company is in one of the heavily polluting industries, the ${\mathrm{Pollu}}_{i}$ value is 1, otherwise it is 0. The Guidelines were issued in 2012, thus the value of ${\mathrm{Policy}}_{t}$ is 1 in 2012 and beyond, otherwise the value is 0. The independent variables are the product of ${\mathrm{Pollu}}_{i}$ and ${\mathrm{Policy}}_{t}$ (hereby referred to as DID).

#### 4.2.3. Control Variables

We introduced the following control variables: enterprise size (denoted by Size), taking total assets to natural logarithm; net margin on total assets (denoted by Roa), which is the net profits divided by the total assets; gearing ratio (denoted by Lev), which is liabilities divided by assets; asset structure (denoted by Coa), which is the current assets divided by the total assets; company growth (denoted by Grow), which is year-on-year growth in operating income; return on net assets (denoted by Roe), which is the net profits divided by average shareholders’ equity.

### 4.3. Descriptive Statistics

The difference in magnitude between the variables is reasonable according to Table 1. The number of observation samples, for which the dependent variable takes the value of 1 in this study, was 5292. This accounts for 27.18% of the total observation samples, which is a reasonable sample selection.

## 5. Empirical Results and Analysis

### 5.1. Test for Parallel Trend

This study conducted empirical tests using the difference-in-differences method, and this method assumes that the dependent variables in both the control group and the treatment group have the same change before the policy implementation. Parallel trend tests were conducted using the visual observation method and the event study method, respectively.

#### 5.1.1. Visual Observation Method

**Figure 1:**
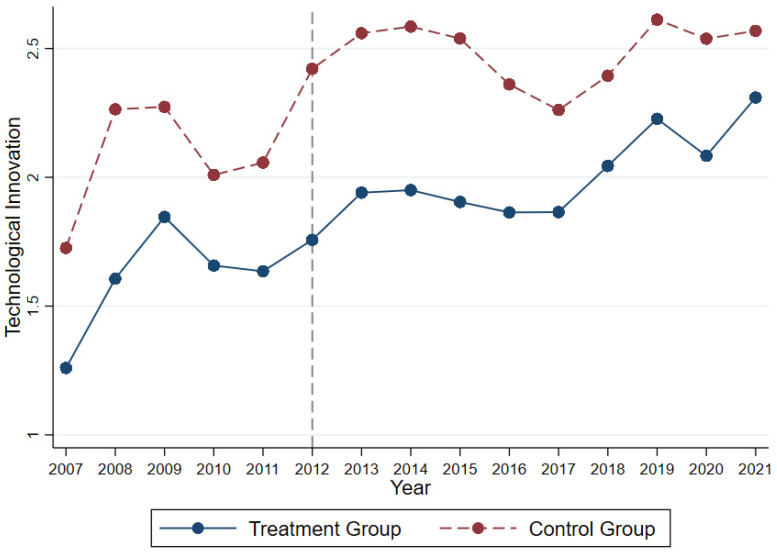
Trend of technological innovation over time.

Figure 1 shows technological innovation over time in the control group and treatment group: Technological innovations in both treatment and control groups show similar changes over time before 2012, which indicates that both groups have no remarkable differences before the implementation of the policy. This means that the sample satisfies the parallel trend assumption.

#### 5.1.2. Event Study Method

**Figure 2:**
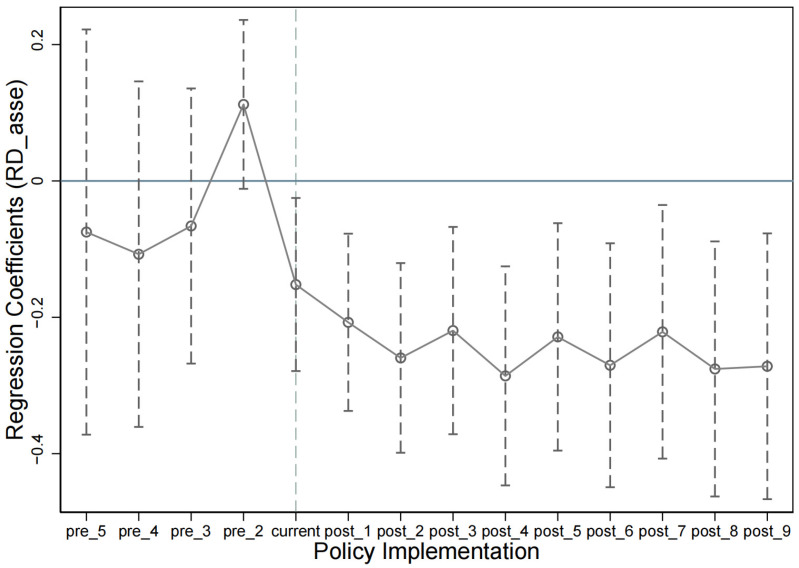
Test for parallel trend and policy dynamic effects.

First, the dummy variable ${\;\mathrm{Tim}}_{t}$ was generated for each year, with ${\;\mathrm{Tim}}_{t}$ having a value of 1 if the year is t, and 0 otherwise. Second, the variable TP was generated through the multiplication of ${\;\mathrm{Tim}}_{t}$ and ${\mathrm{Pollu}}_{i}$. Third, we conducted regression analysis with technological innovation as the dependent variable and TP as the independent variable. Fourth, the coefficients of TP were the differences between the control group and the treatment group in each year. Fifth, the previous period of the policy implementation was used as a reference group to avoid perfect collinearity. Figure 2 shows that the 95% confidence intervals for the regression coefficients of TP all contain zero prior to the implementation of the Guidelines. The results indicated that there is no significant difference between the treatment and the control groups before the policy was implemented, which further supports that the sample satisfies the parallel trend assumption.

As shown in Figure 2, the regression coefficients of TP were significantly negative after the announcement of the Guidelines. This indicates that the technological innovation of companies in the heavily polluting industries was significantly lower than that in other industries as a result of the Guidelines, which initially verified Hypothesis H1.

### 5.2. Results of Baseline Regression 

Table 2 reports the results of the regressions of the Guidelines on technological innovation. Control variables were not included in column (1) but were included in column (2), and both columns controlled for firm fixed effects and year fixed effects. As can be seen from Table 2, the coefficients of DID were significantly negative, which indicates that green finance policies significantly inhibited technological innovation by firms in heavily polluting industries, further confirming Hypothesis H1.

### 5.3. Robustness Tests

We replaced the initial dependent variable with R&D expenditure as a percentage of operating revenue to test robustness. 

**Figure 3:**
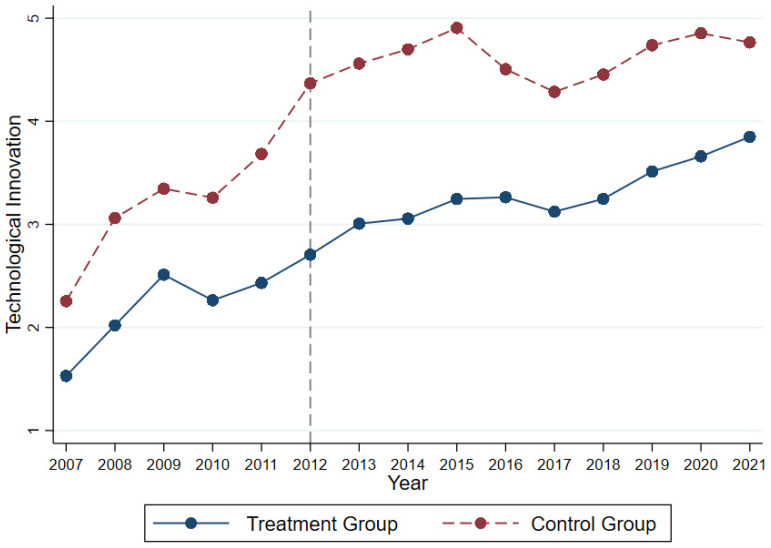
Time trend graph for replacing the dependent variable.

**Figure 4:**
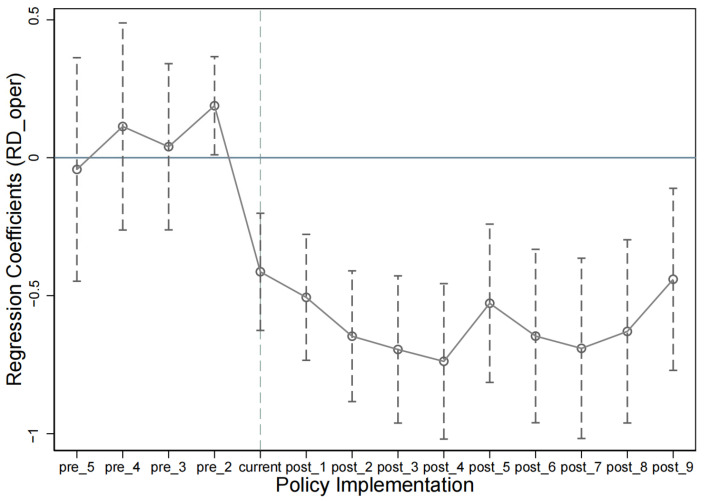
Parallel trend tests for replacing explanatory variables with policy dynamic effects.

As seen in Figure 3 and Figure 4, the new dependent variable largely satisfied the parallel trend hypothesis.

As shown in Table 3, the coefficients of DID were negative, indicating that green financial policies significantly reduced technological innovation, further validating Hypothesis H1.

## 6. Analysis of the Mechanism of Action

We constructed models (2) and (3) to test the mediating roles of bank lending, credit term structure, management motivation, and business confidence.

(2) $$M=\beta_{0}+\beta_{1}{\mathrm{Pollu}}_{i}\times {\mathrm{Policy}}_{t}+\beta_{2}X_{\mathrm{it}}+\varphi_{i}+\varphi_{t}+\varepsilon_{\mathrm{it}}$$

(3) $$\mathrm{RD}\_{\mathrm{asse}}_{\mathrm{it}}=\gamma_{0}+\gamma_{1}{\mathrm{Pollu}}_{i}\times {\mathrm{Policy}}_{t}+\gamma_{2}M+\gamma_{3}X_{\mathrm{it}}+\varphi_{i}+\varphi_{t}+\varepsilon_{\mathrm{it}}$$

where M denotes the mediating variables: bank lending substitution, credit term structure, management motivation, and business confidence, and the remaining variables have the same meaning as in model (1).

The following indicators were selected for the intermediary variables:

- Bank loans: short-term borrowing plus long-term borrowing as a percentage of total assets;
- Credit maturity structure: expressed as long-term borrowing divided by short-term borrowing, with a larger ratio indicating a more long-term credit maturity structure;
- Management motivation: measured using management expenses divided by gross operating revenue, with a higher ratio indicating more active management activity, i.e., more motivated management;
- Business Confidence: measured using intangible assets, cash paid for fixed assets, and other long-term assets as a percentage of total assets, with larger ratios indicating that business decisions tend to be more long-term, reflecting the greater confidence of business decision-makers in the future.

### 6.1. Intermediation Effects of Bank Loans

The regression coefficients of the DID on bank loans without and with the inclusion of control variables were negative (Table 4), indicating that the Guidelines reduced bank loans to heavy polluters. The regression results of bank loans on technological innovation show that the coefficients with and without the inclusion of control variables were both significantly negative. As seen in Table 2, the coefficients of DID were −0.136 and −0.232 when the regression model did not include the bank loan mediator variable but −0.142 and −0.237 after the inclusion of the bank loan mediator variable, indicating that this variable has a ‘masking’ effect. The results indicate that the Guidelines offset part of the decline in technological innovation by discouraging bank lending; in other words, the Guidelines promote technological innovation by reducing bank lending, confirming Hypothesis H2.

The DID coefficient was not significant for the bank loan regression. To ensure the rigour of the study, the Bootstrap test was conducted. Table 5 illustrates the results for a sample of 1000. Bs_1 indicates an indirect mediating effect, which has a significantly negative value, and its 95% confidence interval does not contain zero. Therefore, the mediating effect of bank loans passed the Bootstrap test.

### 6.2. Intermediation Effects of Loan Term

Table 6 reports the regression results of DID on credit maturity structure. It can be seen that the coefficients were negative. This indicates that the Guidelines reduced long-term loans as a proportion of total loans. The regression results of the credit maturity structure on technological innovation show that long-term loans can significantly lead to technological innovation. The regression coefficients of DID on technological innovation were −0.133 and −0.230 when the mediating variable of credit term structure was included. Their absolute values were smaller than the absolute values of −0.136 and −0.232 when the mediating variable was not included. The differences demonstrate the mediating effect. Therefore, the regression results suggest that the Guidelines inhibited technological innovation by reducing the share of long-term credit, confirming Hypothesis H3.

### 6.3. Management Activism Mediating Effects

Table 7 demonstrates the management activism mediation effect. The coefficients of DID on management motivation with and without control variables were significantly negative, indicating that the Guidelines significantly reduced the management motivation of heavy polluters. The regression results of management motivation on technological innovation showed that the coefficients were significantly positive when including and excluding control variables. The regression coefficients of DID on technological innovation were −0.103 and −0.179 when the mediating variable of management motivation was included. Their absolute values were smaller than the absolute values of −0.136 and −0.232 when the control variables were not included. This difference demonstrated the existence of a mediating effect. The above results show that the Guidelines reduced R&D expenditure of companies by discouraging the motivation of corporate management, confirming Hypothesis H4.

### 6.4. Business Confidence Mediating Effects

As shown in Table 8, the coefficients of DID on business confidence when control variables were included and excluded were negative, indicating that the Guidelines reduced business confidence. The regression results of business confidence on technological innovation with and without control variables were significantly positive, indicating that business confidence had a significant contribution to technological innovation. The absolute values of the DID coefficients were 0.131 and 0.229 when the mediating variables were included. These values were smaller than the absolute values of 0.136 and 0.232 when the mediating variables were not included. In summary, the Guidelines significantly inhibited technological innovation by reducing business confidence, confirming Hypothesis H5.

As column (1) in Table 8 was not significant, to enhance the rigour of the study, the Bootstrap method was employed. Table 9 reports the test results. Bs_1 indicates the indirect effect with a negative value, and its 95% confidence interval does not contain zero. The results suggest that business confidence has a mediating effect. 

## 7. Analysis of the Moderating Effect

We constructed model (4) for testing the moderating effects.

(4) $$\mathrm{RD}\_{\mathrm{asse}}_{\mathrm{it}}=\theta_{0}+\theta_{1}{\mathrm{Pollu}}_{i}\times {\mathrm{Policy}}_{t}+\theta_{2}{\mathrm{Pollu}}_{i}\times {\mathrm{Policy}}_{t}\times \mathrm{Turn}+\theta_{3}X_{\mathrm{it}}+\varphi_{i}+\varphi_{t}+\varepsilon_{\mathrm{it}}$$

where Turn indicates the operating capacity of a company. It was measured by the total asset turnover ratio. We observed the product of ${\mathrm{Pollu}}_{i}$ × ${\mathrm{Policy}}_{t}$ × Turn (denoted by DID × Turn in the table below).

As shown in Table 10, the DID × Turn was significantly positive, indicating that firm operating capacity can significantly weaken the inhibitory effect of green finance, and thus confirmed Hypothesis H6.

## 8. Conclusions

In this study, multiple effects of green financial policies were analysed from three perspectives: compliance cost effect, innovation compensation effect, and policy signalling effect. The mediating and moderating effects of green financial policies on technological innovation were also analysed. Furthermore, an empirical test was conducted using the difference-in-differences method. The following conclusions were drawn. First, green finance inhibited technological innovation owing to the pessimistic attitude of heavily polluting firms towards the returns expected from technological innovation. Second, green financial policies weakened the constraints of banks on technological innovation by lowering bank loans, and thus promoted technological innovation. Third, green financial policies resulted in shorter credit terms, lower management incentives, and weaker business confidence, and thus discouraged technological innovation. Fourth, the stronger the operating capacity of enterprises, the weaker the policy’s inhibiting effect on technological innovation.

Accordingly, the following recommendations are made for improving green financial policies and using technological innovation to achieve green growth. First, avoid the wholesale rejection of heavily polluting industries and emphasise green transformation and upgrading. It is not reasonable to shut down heavily polluting industries across the board; therefore, guiding enterprise transformation is a necessary step to combat environmental pollution while considering economic development. The emphasis on transformation and upgrading can give confidence to the industry while curbing the blind expansion and sloppy development of heavily polluting industries. This can guide market players to be proactive rather than passive and motivate enterprises to address the contradiction between maximising profits and environmental constraints through technological innovation. Second, banks should be guided to adjust their credit maturity structure for enterprises in heavily polluting industries. Owing to their own interests, banks lack the incentive to extend the credit maturity of enterprises in heavily polluting industries. Therefore, the green financial policy should strengthen the guidance and constraint for banks to increase the proportion of long-term loans to provide stable financial security for R&D and innovation. Finally, banks should be guided to reduce their intervention in the normal operation of enterprises while strengthening their environmental review. Banks’ loan reviews and interventions can impact the normal business activities of enterprises. Green financial policies should guide and restrain the behaviour of banks to avoid excessive intervention in business activities [31,32,33,34].

In the study, we analysed the significance of technological innovation and green finance with regards to promoting green growth and reducing environmental pollution, as well as their relationships. However, this study also had some limitations. First, this study was based on the Green Credit Guidelines from China, and different countries have different green financial policies; therefore, the applications of the findings of this study to other countries require further analysis. Second, owing to word limit, this study did not specifically analyse the heterogeneous role of technological innovation in different industries. Further research can be done in the future in the above two respects.

## Figures and Tables

**Table 1:** Variable descriptions.

Vars	Obs	Mean	SD	Min	Max
RD_asse	19,469	2.252	1.792	0.036	11.998
Size	19,469	21.914	1.286	18.187	26.301
Lev	19,469	43.639	18.176	5.716	94.332
Roe	19,469	11.337	9.727	−49.241	62.791
Roa	19,469	6.228	5.180	−20.188	34.427
Coa	19,469	57.474	18.098	10.202	97.179
Grow	19,469	19.760	41.036	−86.248	1773.843

**Table 2:** Baseline regression results.

	(1)	(2)
Vars	RD_asse	RD_asse
DID	−0.136 *	−0.232 ***
	(−1.80)	(−3.39)
Size		−0.608 ***
		(−15.04)
Lev		0.009 ***
		(6.44)
Roe		0.007 *
		(1.81)
Roa		0.041 ***
		(4.93)
Coa		0.002 *
		(1.86)
Grow		0.000
		(−2.36)
Firm FE	Yes	Yes
Year FE	Yes	Yes
Constant	1.674 ***	13.090 ***
	(20.46)	(15.37)
Observations	19,469	19,469
R-Square	0.035	0.153
adj. R-Square	0.034	0.153

Notes: * and *** denote significance levels, which are 10% and 1%, respectively. The *t* statistics are in parentheses.

**Table 3:** Results of robustness test.

	(1)	(2)
Vars	RD_oper	RD_oper
DID	−0.597 ***	−0.655 ***
	(−5.25)	(−5.77)
Size		−0.145 *
		(−1.84)
Lev		−0.020 ***
		(−7.95)
Roe		0.021 ***
		(2.91)
Roa		−0.066 ***
		(−4.46)
Coa		−0.005 *
		(−1.82)
Grow		−0.003 ***
		(−6.01)
Firm FE	Yes	Yes
Year FE	Yes	Yes
Constant	2.310 ***	6.914 ***
	(23.13)	(4.22)
Observations	19,469	19,469
R-Square	0.112	0.135
adj. R-Square	0.111	0.134

Notes: * and *** denote significance levels, which are 10% and 1%, respectively. The *t* statistics are in parentheses.

**Table 4:** Tests of intermediation effects of bank loans.

	(1)	(2)	(3)	(4)
Vars	Loan	Loan	RD_asse	RD_asse
DID	−2.002 ***	−0.588	−0.142 *	−0.237 ***
	(−3.58)	(−1.56)	(−1.88)	(−3.45)
Loan			−0.003 *	−0.008 ***
			(−1.80)	(−3.95)
Control variables	No	Yes	No	Yes
Firm FE	Yes	Yes	Yes	Yes
Year FE	Yes	Yes	Yes	Yes
Constant	20.070 ***	18.770 ***	1.732 ***	13.240 ***
	(32.78)	(3.92)	(19.79)	(15.69)
Observations	19,469	19,469	19,469	19,469
R-Square	0.037	0.518	0.035	0.155
adj. R-Square	0.036	0.517	0.035	0.155

Notes: * and *** denote significance levels, which are 10% and 1%, respectively. The *t* statistics are in parentheses.

**Table 5:** Bootstrap test for the intermediation effect of bank loans.

	Observed	Bootstrap			Normal Based	
	Coef.	Std. Err.	z	P > |z|	[95% Conf. Interval]	
Bs_1	−0.034	0.004	−8.850	0.000	−0.041	−0.026
Bs_2	−0.033	0.026	−1.250	0.213	−0.085	0.019

**Table 6:** Test for the mediating effect of loan term.

	(1)	(2)	(3)	(4)
Vars	Term	Term	RD_asse	RD_asse
DID	−0.852 **	−0.669 *	−0.133 *	−0.230 ***
	(−2.22)	(−1.77)	(−1.77)	(−3.36)
Term			0.003 *	0.003 *
			(1.84)	(1.86)
Control variables	No	Yes	No	Yes
Firm FE	Yes	Yes	Yes	Yes
Year FE	Yes	Yes	Yes	Yes
Constant	−4.899 ***	−26.850 ***	1.689 ***	13.160 ***
	(−10.92)	(−6.68)	(20.54)	(15.48)
Observations	19,469	19,469	19,469	19,469
R-Square	0.077	0.091	0.035	0.154
adj. R-Square	0.076	0.090	0.035	0.153

Notes: *, **, and *** denote significance levels, which are 10%, 5%, and 1%, respectively. The *t* statistics are in parentheses.

**Table 7:** Test of the mediating effect of corporate management motivation.

	(1)	(2)	(3)	(4)
Vars	Admi	Admi	RD_asse	RD_asse
DID	−0.739 ***	−0.831 ***	−0.103	−0.179 ***
	(−4.33)	(−5.18)	(−1.36)	(−2.63)
Admi			0.045 ***	0.064 ***
			(7.93)	(10.31)
Control variables	No	Yes	No	Yes
Firm FE	Yes	Yes	Yes	Yes
Year FE	Yes	Yes	Yes	Yes
Constant	7.738 ***	31.480 ***	1.328 ***	11.060 ***
	(49.00)	(11.54)	(14.17)	(12.82)
Observations	19,469	19,469	19,469	19,469
R-Square	0.084	0.202	0.050	0.181
adj. R-Square	0.084	0.201	0.049	0.180

Notes: *** denote significance levels, which are 1%. The *t* statistics are in parentheses.

**Table 8:** Test of mediating effects of business confidence.

	(1)	(2)	(3)	(4)
Vars	Inve	Inve	RD_asse	RD_asse
DID	−0.441	−0.446 *	−0.131 *	−0.229 ***
	(−1.63)	(−1.73)	(−1.74)	(−3.36)
Inve			0.011 ***	0.006 **
			(4.52)	(2.47)
Control variables	No	Yes	No	Yes
Firm FE	Yes	Yes	Yes	Yes
Year FE	Yes	Yes	Yes	Yes
Constant	9.301 ***	18.930 ***	1.571 ***	12.980 ***
	(30.4)	(6.38)	(18.18)	(15.27)
Observations	19,469	19,469	19,469	19,469
R-Square	0.109	0.164	0.037	0.154
adj. R-Square	0.109	0.164	0.036	0.153

Notes: *, **, and *** denote significance levels, which are 10%, 5%, and 1%, respectively. The *t* statistics are in parentheses.

**Table 9:** Bootstrap test of the mediating effect of business confidence.

	Observed	Bootstrap			Normal Based	
	Coef.	Std. Err.	z	P > |z|	[95% Conf. Interval]	
Bs_1	−0.010	0.002	−4.830	0.000	−0.014	−0.006
Bs_2	−0.057	0.025	−2.270	0.023	−0.105	−0.008

**Table 10:** Test of the moderating effect of firm operating capacity.

	(1)	(2)
Vars	RD_asse	RD_asse
DID	−0.920 ***	−0.716 ***
	(−8.88)	(−7.49)
DID×Turn	1.096 ***	0.681 ***
	(10.92)	(7.00)
Control variables	No	Yes
Firm FE	Yes	Yes
Year FE	Yes	Yes
Constant	1.676 ***	12.920 ***
	(20.6)	(15.27)
Observations	19,469	19,469
R-Square	0.054	0.161
adj. R-Square	0.053	0.160

Notes: *** denote significance levels, which are 1%. The *t* statistics are in parentheses.

## Data Availability

The data of this study are available from the corresponding author upon reasonable request.
